# Long-term clinical outcomes and prognostic factors of upfront surgery as a first-line therapy in biopsy-proven clinical N2 non-small cell lung cancer

**DOI:** 10.3389/fonc.2022.933278

**Published:** 2022-07-28

**Authors:** Luca Bertolaccini, Elena Prisciandaro, Juliana Guarize, Lara Girelli, Giulia Sedda, Niccolò Filippi, Filippo de Marinis, Lorenzo Spaggiari

**Affiliations:** ^1^ Department of Thoracic Surgery, IEO, European Institute of Oncology IRCCS, Milan, Italy; ^2^ Unit of Interventional Pneumology, IEO, European Institute of Oncology IRCCS, Milan, Italy; ^3^ Department of Thoracic Oncology, IEO, European Institute of Oncology IRCCS, Milan, Italy; ^4^ Department of Oncology and Hemato-Oncology, University of Milan, Milan, Italy

**Keywords:** lung cancer, multimodal treatment, induction chemotherapy, upfront surgery, thoracic surgery

## Abstract

**Background:**

Multimodality therapy offers the best opportunity to improve pathological N2 non-small cell lung cancer (NSCLC) prognosis. This paper aimed to evaluate the long-term clinical outcomes and the prognostic factors of upfront surgery as first-line therapy in biopsy-proven clinical N2.

**Methods:**

Retrospective review of biopsy-proven cN2 NSCLC patients operated between 2007 and 2017. Upfront surgery was considered if the primary tumour was deemed completely resectable, with mediastinal nodal involvement confined to a single station and no preoperative evidence of extranodal tumour invasion.

**Results:**

Two hundred eighty-five patients who underwent radical resections were included. One hundred fifty-nine patients (55.8%) received induction chemotherapy. At follow-up completion, 127 (44.6%) patients had died. For the induction chemotherapy group, the median overall survival (OS) was 49 months [95% confidence interval (CI): 38–70 months], and the 5-year OS was 44.4%. The median and 5-year OS for the up front surgery group was 66 months (95% CI: 40–119 months) and 66.3%, respectively. There were no statistically significant differences between treatment approaches (p = 0.48). One hundred thirty-four patients (47.0%) developed recurrence. The recurrence-free survival (RFS) at 5 years was 17% (95% CI: 11–25%) for induction chemotherapy and 22% (95% CI: 9–32%) for upfront surgery; there were no statistically significant differences between groups (p = 0.93). No significant differences were observed based on the clinical N status (OS, p = 0.36; RFS, p = 0.65).

**Conclusions:**

Upfront surgery as first-line therapy for biopsy-proven cN2 NSCLC showed favourable clinical outcomes, similar to those obtained after induction chemotherapy followed by surgery. Therefore, it should be considered one of the multimodality treatment options in resectable N2 NSCLC.

## Introduction

Stage IIIA/N2 NSCLC patients are a heterogeneous population with a broad range of clinical presentations (1). The wide variety of features and manifestations among N2 patients is reflected in the non-univocity of the possible treatment approaches and management strategies. For simplicity, N2 patients have been classified into three distinct subgroups. Patients with *occult* or *unforeseen N2 disease* do not show lymph node involvement at preoperative staging (clinical N0, cN0) but are diagnosed with N2 disease at pathological analysis postoperatively; they typically receive adjuvant therapies following the complete surgical resection. The second category includes unresectable tumours with *bulky N2 involvement*, whose treatment is definitive chemoradiotherapy. The third subgroup (*possibly resectable N2 disease*) shows pathologically proven mediastinal lymph node involvement at preoperative clinical staging (2). The presence of pathological N2 (pN2) nodes implies a significant probability of systemic relapse; in this perspective, multimodality therapies offer the best chance of improving pN2 disease prognosis (3). However, due to the aforementioned N2 disease clinical variability, selecting the best combination and sequence of multimodality treatments for pN2 disease remains one of the most complex issues in clinical practice (3). Although surgical resection is still debatable for the third group of patients, the outcomes obtained following the first surgery or radiation alone prompted consideration of induction chemotherapy to improve resectability to improve long-term overall survival (OS) rates (2).

On the other hand, upfront surgical resection followed by adjuvant therapy is currently one of the therapeutical options for resectable N2 disease. For a subset of patients with pIIIA NSCLC, surgery followed by adjuvant therapy may yield positive survival outcomes, particularly for individuals with AIDS or clinical N0 and pathologic single-station N2 malignancies (4). For resectable N2, the outcomes of surgery first followed by adjuvant therapy were superior to those reported in prior studies. In order to improve the prognosis of patients undergoing initial surgery for N2, adjuvant chemotherapy is needed (3).

This work aimed to evaluate the long-term clinical outcomes and the prognostic factors of upfront surgery as first-line therapy in biopsy-proven N2 disease.

## Material and methods

Between January 2007 and December 2017, we retrospectively analysed the consecutive records of patients with biopsy-proven N2 NSCLC from a prospectively acquired institutional database. Patients with concurrent malignancies, patients who received neoadjuvant therapy, patients who underwent incomplete surgical resection resections, and patients with missing data were excluded from this study. The Ethics Committee and the Internal Review Board, informed of the database extraction, did not require approval because of the study’s retrospective nature. This manuscript was written according to the Strengthening the Reporting of Cohort Studies in Surgery (STROCSS) Statement (5). The STROCSS checklist is available as **Supplemental File 1**.

**Figure 1:**
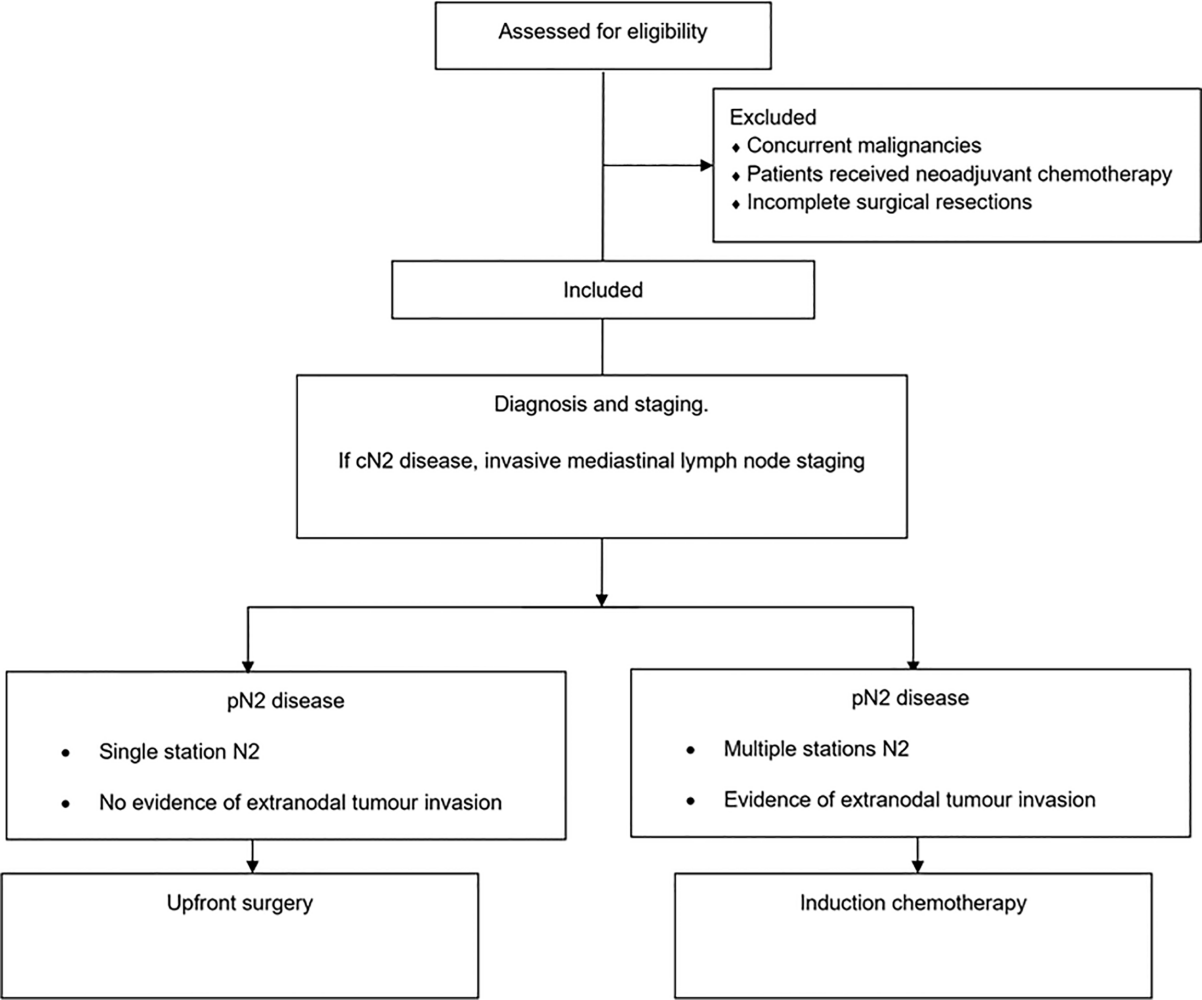
Flowchart with the study design.

The diagnosis and staging for patients with NSCLC followed well-established, widely accepted protocols (6–9). When the computed tomography (CT) scan and/or 18-fluoro-deoxy-glucose positron emission tomography (^18^FDG-PET) scan revealed cN2 disease, invasive mediastinal lymph node staging was performed through endobronchial ultrasonography-guided transbronchial needle aspiration, oesophageal ultrasonography-guided transoesophageal needle aspiration, and/or mediastinoscopy (10). The multidisciplinary team devised treatment strategies for pathologically confirmed N2 NSCLC patients. Upfront surgery for pathologically proven N2 disease was generally considered when mediastinal lymph node involvement was limited to a single station without evidence of extranodal tumour invasion (11, 12). All patients underwent lung resection followed by systematic radical lymphadenectomy (**Figure 1**). Duration of surgical procedure was defined as the duration from the first skin incision to final wound closure, expressed in minutes. Operative mortality was defined as deaths occurring within 30 days of surgery or during the same hospitalisation period regardless of aetiology. Pathological staging was performed according to the American Joint Committee on Cancer 8th edition standards (1). One hundred forty-five patients with pN2 disease underwent adjuvant chemotherapy, except those aged older than 75 or in poor physical condition, as determined by the multidisciplinary team; the medical oncologist decided on the chemotherapy regimen according to the current guidelines—complications were classified using the Clavien–Dindo classification (13). Besides, postoperative hospital length of stay was employed as a surrogate marker of postoperative morbidity.

### Statistical analysis

The mean and standard deviation (SD) of quantitative variables were used, while nominal variables were presented as presence or absence of the occurrence. The Kruskal–Wallis rank test was employed for continuous variables, and for categorical variables, the Fisher exact test was utilised. The time gap between operation and death was defined as the OS. The time interval between resection and disease relapse was defined as the recurrence-free survival (RFS), and patients without recurrence were censored at the latest time known to be recurrence-free. The reverse Kaplan–Meier approach calculated the median OS and RFS. The median OS, hazard ratio (HR), and 95% confidence intervals (CI) were used to describe differences in survival rates, and the log-rank test was used to compare them. Bonferroni correction was applied for multiple comparisons, and a p-value of less than 0.05 was considered significant. The standard, *EZR, irr, rcmdr*, and *ROC* packages were used in RStudio (R version 4.1.0, Camp Pontanezen) for statistical analysis (14, 15).

## Results

**Figure 2:**
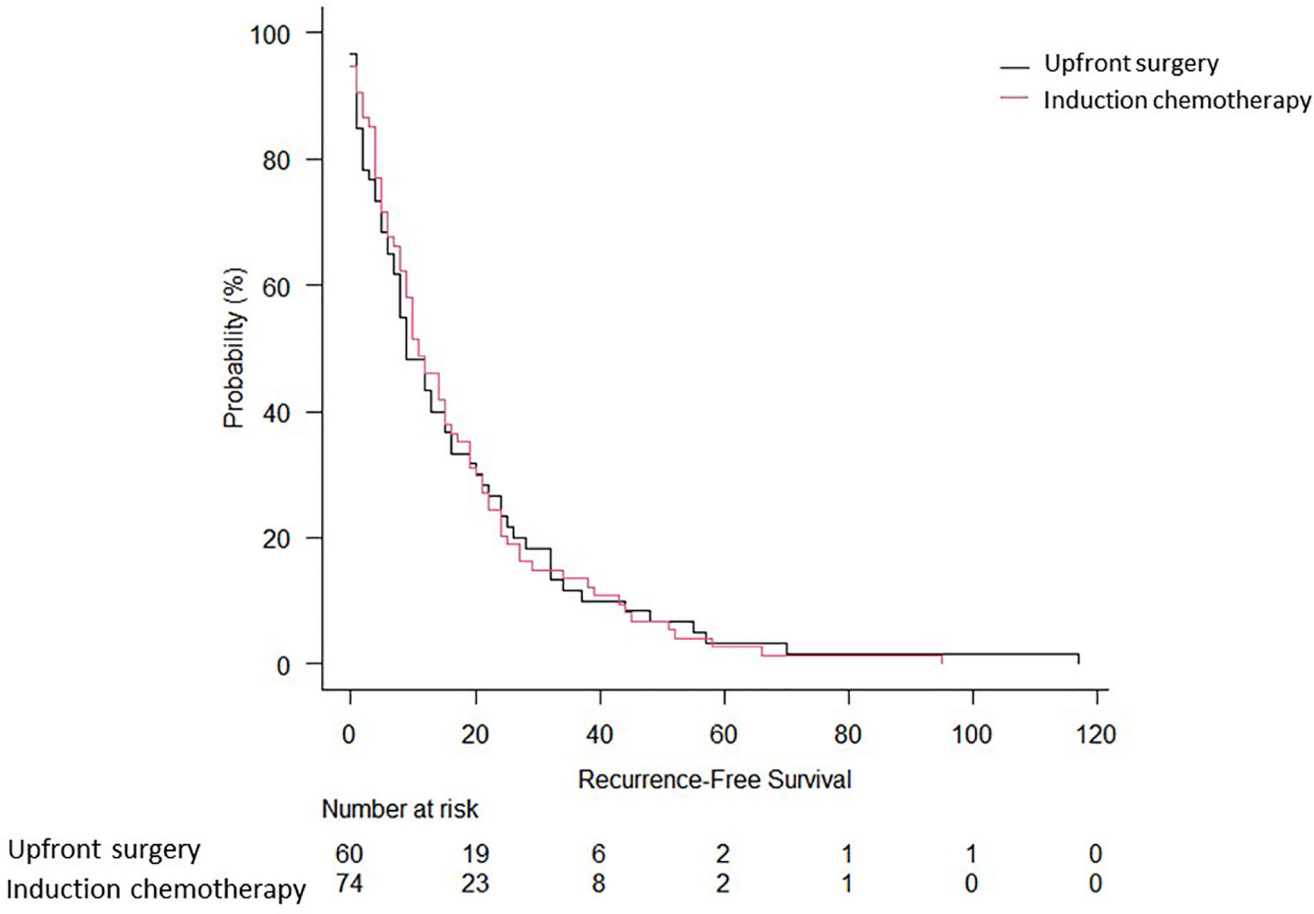
Overall survival of biopsy-proven N2 NSCLC patients who underwent upfront surgery or induction chemotherapy.

**Figure 3:**
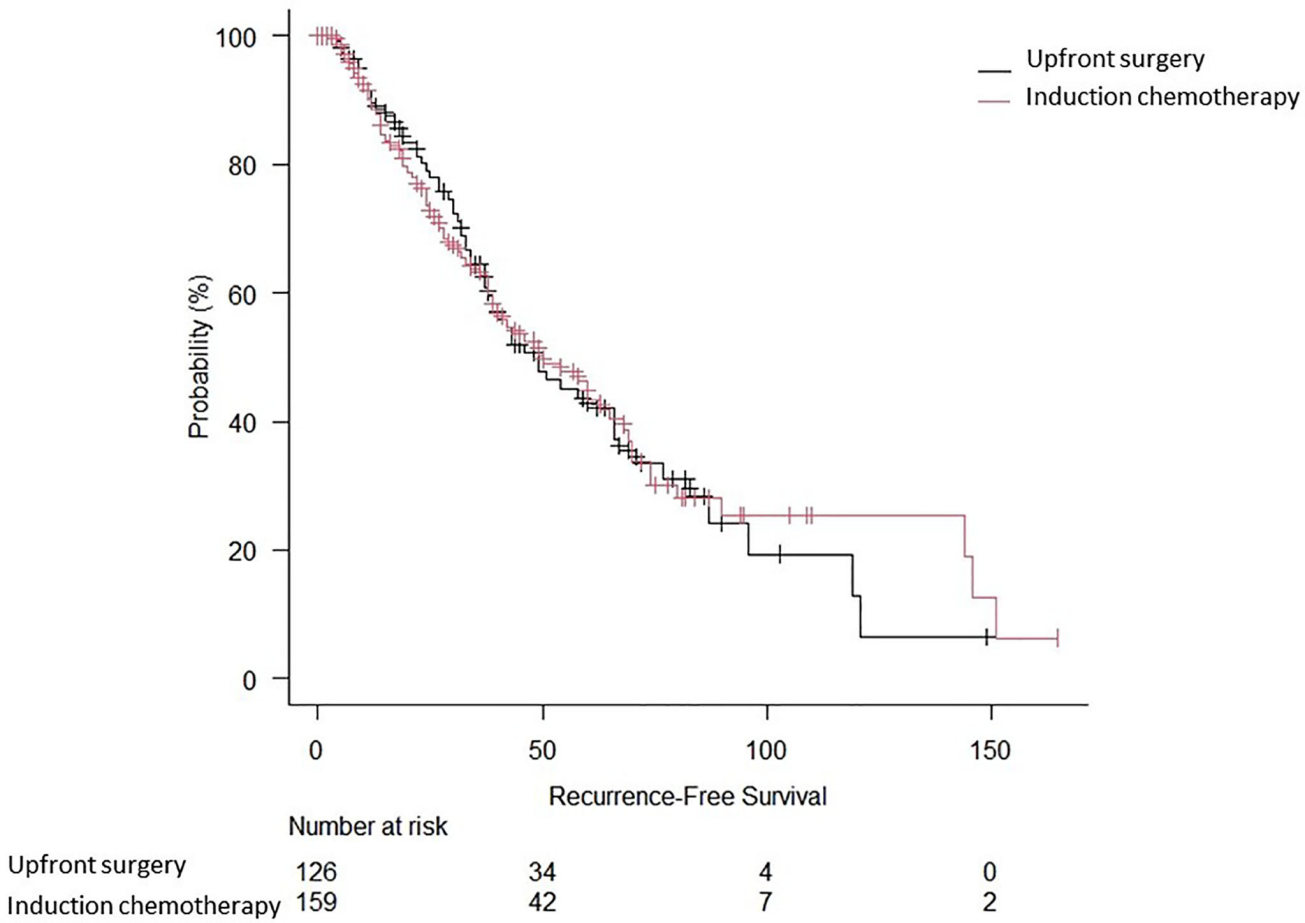
Recurrence-free survival of biopsy-proven N2 NSCLC patients who underwent upfront surgery or induction chemotherapy.

**Figure 4:**
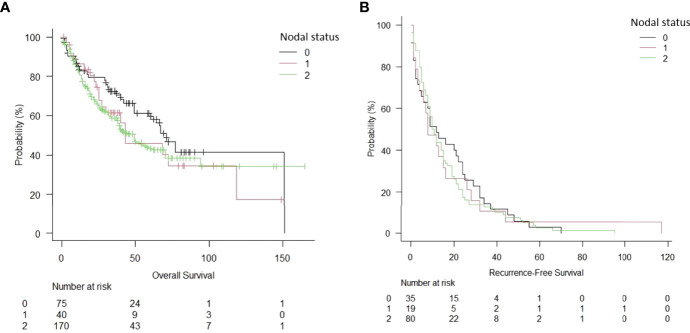
Overall survival **(A)** and recurrence-free survival **(B)** of NSCLC patients, depending on the nodal status.

Two-hundred eighty-five patients who underwent lung resection for NSCLC at our institution were included during the study period. The main characteristics of the included patients are summarised in **Table 1**. Induction therapy was administered to 159 patients (55.8%). Operative mortality was 0.35% (n = 2). The preoperative and intraoperative characteristics, as well as the pathological staging of the study population, are summarised in **Table 2**. The surgical approach was mainly thoracotomic (n = 255 [89.5%]). There were no significant differences between the two groups regarding the American Society of Anaesthesiology (ASA) score, duration of surgical procedure, and postoperative length of stay. Overall complications are reported in **Table 3**. The mean follow-up time was 39.6 ± 24.7 months. At follow-up completion, 127 patients (44.6%) had died, while the remaining 158 (55.4%) were living or censored. The median OS was 49 months [95% confidence interval (CI): 38–70 months], and the 5-year OS was 44.4% in the induction chemotherapy group. The median and 5-year OS for the upfront surgery group was 66 months (95% CI: 40–119) and 66.3%, respectively (**Figure 2**). Hence, there were no statistically significant differences between the two treatment approaches (p = 0.48). One hundred thirty-four patients (47.0%) developed recurrence. The RFS at 5 years was 17% (95% CI: 11–25) for induction chemotherapy and 22% (95% CI: 9–32%) for upfront surgery (**Figure 3**). There were no statistically significant differences between the two groups (p = 0.93). Based on the clinical N status (**Figure 4**), no significant differences were observed in terms of OS (p = 0.36) and RFS (p = 0.65).

**Table 1:** Demographic characteristics of the study population.

	Induction chemotherapy (N = 159)	Upfront surgery (N = 126)	*p-value*
Age (years), mean ± SD	62.7 ± 9.0	66.3 ± 8.3	*0.58*
Sex, number of patients (%)			*0.74*
* Male	104 (65.4)	80 (63.5)	
* Female	55 (74.6)	46 (36.5)	
Main comorbidities, number of patients (%)			*0.51*
* Cardiac	66 (41.5)	73 (57.9)	
* Pulmonary	14 (8.8)	20 (15.9)	
Pulmonary function tests, mean ± SD			*0.96*
* FEV1%	88.1 ± 18.9	93.5 ± 20.3	
* DLCO/VA	82.9 ± 20.6	90.4 ± 20.7	
Histology, number of patients (%)			*0.54*
* Adenocarcinoma	120 (75.5)	105 (83.3)	
* Squamous cell carcinoma	31 (19.5)	16 (12.7)	
* Adenosquamous	4 (2.5)	5 (4.0)	
* Neuroendocrine	3 (1.9)	0	
* Atypical carcinoid	1 (0.6)	0	

DLCO/VA, diffusing capacity of carbon monoxide divided by the alveolar volume; FEV1%, forced expiratory volume in the first second (measured/predicted %); SD, standard deviation.

**Table 2:** Preoperative and intraoperative characteristics and pathological analysis of the study population.

	Induction chemotherapy (N = 159)	Upfront surgery (N = 126)	*p-value*
ASA score, number of patients (%)			*0.80*
* 1	3 (1.9)	2 (1.6)	
* 2	111 (69.8)	82 (65.1)	
* 3	44 (27.7)	42 (33.3)	
* 4	0	0	
Surgical approach, number of patients (%)			*0.026*
* Thoracotomy * VATS * Robot	155 (97.5) 0 4 (2.5)	100 (79.4) 11 (8.7) 15 (11.9)	
Type of lung resection, number of patients (%)			*0.0038*
* Anatomical segmentectomy * Lobectomy * Bilobectomy * Pneumonectomy	2 (1.3) 88 (55.3) 13 (8.2) 56 (35.2)	3 (2.4) 93 (73.8) 7 (5.6) 23 (18.2)	
Duration of surgery (min), median (range)	174.0 (75–732)	167.5 (76–427)	*0.83*
Pathological staging (1), number of patients (%)			*0.66*
* IIIA * IIIB	93 (58.5) 66 (41.5)	77 (61.1) 49 (38.9)	

ASA, American Society of Anaesthesiology; VATS, video-assisted thoracic surgery.

**Table 3:** Overall postoperative complications of the study population.

	Induction chemotherapy(N = 159)	Upfront surgery(N = 126)	*p-value*
Postoperative length of stay (days), median (range)	6 (4–15)	6 (4–33)	*0.93*
Overall complications, number of events (%)	75 (47.2)	41 (32.5)	*0.74*
Complications according to Clavien–Dindo classification(13), number of events (%)			*0.12*
* 0 * 1 * 2 * 3a * 3b * 4a * 4b * 5	84 (52.2) 15 (9.4) 38 (23.9) 6 (3.7) 7 (4.4) 3 (1.9) 2 (1.3) 4 (2.5)	85 (67.5) 14 (11.1) 16 (12.7) 6 (4.8) 2 (1.6) 2 (1.6) 0 1 (0.8)	

## Discussion

NSCLC accounts for more than 85% of all lung cancer histologies, and roughly one-third of NSCLC cases are diagnosed at locally advanced stages (9). The usual treatment for suitably selected patients with locally advanced disease (stage IIIA and IIB) implies a multidisciplinary approach that includes total surgical resection, chemotherapy, radiation, targeted therapy, and immunotherapy (16). Since the 1990s, chemotherapy has been the primary treatment for advanced NSCLC, resulting in numerous trials in the neoadjuvant context for early-stage NSCLC. Indeed, these data emphasise the need to track adverse events throughout treatment. Luckily, the development of immunotherapy has ushered in a new era in lung cancer treatment. Even when used alone, immune checkpoint inhibitors have considerable advantages over chemotherapy in stage IV NSCLC (17).

The optimal chemotherapy timing (induction versus adjuvant treatment) for individuals with N2 lymph nodes is unclear. Therefore, if a radical (R0) resection is deemed feasible, patients with pN2 disease can benefit from surgery with minimal morbidity and mortality. There is a risk of disease progression following neoadjuvant treatment, which may exclude the prospect of surgical resection (3). Pulmonary resection following neoadjuvant immunotherapy or chemo-immunotherapy for resectable NSCLC appears safe in the current population, with a low risk of operative mortality and morbidity. Current data indicate that the complexity of the operation is comparable to that of patients treated with other neoadjuvant medications (16).

The presence of lymph nodal tumour involvement is a robust predictor of local control and overall survival in patients with NSCLC. A higher number of positive lymph nodes is associated with a poor outcome (18). The eighth lung cancer tumour, node, and metastasis (TNM) classification only considers the anatomical site of lymph nodal stations for lymph node testing. However, merely locating positive lymph node stations is insufficient to provide an accurate estimate of survival (19). In particular, the number of lymph node stations involved seems to affect survival and the anatomic location of the single-level positive lymph nodes. Significantly worse survival rates were observed in multilevel N2 patients compared to single-station N2 patients. Patients with inferior positive mediastinal N2 nodes appear to have the same OS and PFS as patients with multilevel N2 lymph nodes (19).

The published research indicates that lymphatic outflow from each lobe often follows a well-defined anatomic path. This fixed lymphatic drainage channel concept is the foundation upon which lobe-specific lymphadenectomy is performed. Upper lobe tumours often drain to the upper mediastinal lymph nodes (stations 2–6), while middle and lower lobe tumours drain to the lower mediastinal lymph nodes (stations 7–9) (20). Also, it has been shown that patients with right upper lobe NSCLC are more likely to develop skip metastases since direct lymphatic drainage from segmental lymph nodes to mediastinal lymph nodes occurs more frequently (21).

Adjuvant chemotherapy is critical for preventing distant metastases following complete resection of the tumour, particularly in patients with N2 disease who undergo upfront surgery. Nonetheless, no study has motivated the prognosis improvement of this subset of patients (3).

Although patients with unsuspected pN2 disease treated with pulmonary lobectomy have a worse OS than those with cT1–T3 cN2 disease treated with induction therapy followed by surgery, a matched analysis revealed no significant difference in survival between patients with unsuspected pN2 disease and those with suspected N2 disease. Additionally, patients with undetected N2 lymph nodes who undergo lobectomy followed by adjuvant chemotherapy with or without radiation have a better prognosis than those who receive adjuvant radiation alone or with no adjuvant therapy. Thus, lobectomy is appropriate for undetected pN2 lymph nodes if the patient is likely to tolerate adjuvant chemotherapy with or without radiation therapy (22). The extent of resection significantly affects the OS, and patients resectable with lobectomy or pneumonectomy had a significantly longer median OS than those who required extended resections. Regrettably, the necessity for an extended resection is occasionally discovered intraoperatively after hilar structures have been separated (23).

In the Checkmate 816 clinical study, 24% of patients treated with nivolumab and chemotherapy achieved a complete pathological response. In patients with resectable NSCLC, nivolumab with chemotherapy resulted in considerably longer event-free survival and a more significant proportion of patients with a complete histological response than chemotherapy alone. The addition of nivolumab to neoadjuvant chemotherapy did not increase the frequency of adverse events or hinder the surgical viability of patients (24).

In the Pacific study, consolidation durvalumab was linked with substantial and sustained OS and durable PFS benefits following chemoradiation. At 5 years, an estimated 42.9% of patients randomly assigned to durvalumab are still alive, and 33.1% of patients randomly assigned to durvalumab are still living and disease-free, establishing a new standard of treatment in this setting (25).

Lastly, a favourable economic implication of upfront surgery could be found. With the same outcomes, it is advisable to choose the less expensive option (26).

## Limitations

This study had some limitations, mainly inherent to its retrospective and single-centre nature. This is a cohort study; therefore, the discrepancies in baseline patient information should also be considered when comparing our findings to other published research. In addition, patient selection for induction chemotherapy may have introduced selection bias. Finally, resectable N2 lymph nodes are not quantifiable, and the surgeon’s experience may impact the radicality of lymphadenectomy. To address these constraints, prospective, multicentre, randomised controlled trials will be required.

## Conclusions

Upfront surgery as first-line therapy for biopsy-proven N2 NSCLC showed favourable clinical outcomes, similar to those obtained after induction chemotherapy followed by surgery. Adjuvant chemotherapy is critical to improving the prognosis of this subset of patients. Therefore, upfront surgery followed by adjuvant chemotherapy should be considered a valid multimodal therapeutic option in resectable N2 NSCLC.

## Data availability statement

The datasets generated and analysed during the current study are available from the corresponding author on reasonable request.

## Ethics statement

Ethical review and approval was not required for the study on human participants in accordance with the local legislation and institutional requirements. The patients/participants provided their written informed consent to participate in this study.

## Author contributions

All authors listed have made a substantial, direct, and intellectual contribution to the work and approved it for publication.

## Funding

This work was partially supported by the Italian Ministry of Health with *Ricerca Corrente* and *5x1000* funds.

## Conflict of interest

The authors declare that the research was conducted in the absence of any commercial or financial relationships that could be construed as a potential conflict of interest.

## Publisher’s note

All claims expressed in this article are solely those of the authors and do not necessarily represent those of their affiliated organizations, or those of the publisher, the editors and the reviewers. Any product that may be evaluated in this article, or claim that may be made by its manufacturer, is not guaranteed or endorsed by the publisher.
